# Development of a Novel Retina−Based Diagnostic Score for Early Detection of Major Depressive Disorder: An Interdisciplinary View

**DOI:** 10.3389/fpsyt.2022.897759

**Published:** 2022-05-19

**Authors:** Xiao Liu, Shunkai Lai, Shisi Ma, Hong Yang, Lian Liu, Guocheng Yu, Shuming Zhong, Yanbin Jia, Jingxiang Zhong

**Affiliations:** ^1^Department of Ophthalmology, The First Affiliated Hospital of Jinan University, Guangzhou, China; ^2^Department of Psychiatry, The First Affiliated Hospital of Jinan University, Guangzhou, China; ^3^Department of Ophthalmology, The Sixth Affiliated Hospital of Jinan University, Dongguan, China

**Keywords:** major depressive disorder, retina, optical coherence tomography angiography, vessel density, visual field, choroidal thickness

## Abstract

**Background:**

Clinically effective markers for the diagnosis of major depressive disorder (MDD) are lacking. Alterations in retinal features are closely related to the pathophysiological progression of MDD. However, the reliable retina-related diagnostic model for MDD remains to be developed. Thus, our study aimed to quantitatively evaluate retinal vascular and structural changes in MDD patients and to develop a reliable diagnostic model of MDD based on retinal parameters.

**Methods:**

Seventy-eight patients with MDD and 47 healthy controls (HCs) underwent retinal vessel density and structure examination using optical coherence tomography angiography and visual field examination using perimetry. Independent-sample *t* test was used to assess the differences in retinal parameters between the groups. Meanwhile, we constructed the corresponding retina-based diagnostic model by LASSO logistic regression. Finally, the diagnostic ability of the model was evaluated by area under the curve (AUC) of receiver operating characteristic curves and calibration plot of nomogram.

**Results:**

MDD patients showed lower retinal vessel density (including radial peripapillary capillary vessel density, superficial and deep capillary plexus vessel density), thinner subfoveal choroidal thickness, and poorer visual fields compared to HCs (all *p* < 0.05). Furthermore, a retina-based diagnostic model was constructed and shows a strong diagnostic capability for MDD (AUC = 0.9015, *p* < 0.001).

**Conclusion:**

Patients with MDD showed distinct retinal features compared to HCs. The retina-based diagnostic model is expected to be a necessary complement to the diagnosis of MDD.

## Introduction

Major depressive disorder (MDD) is a psychiatric disorder characterized primarily by depressed mood and anhedonia. With a lifetime prevalence of 16%, MDD is one of the leading causes of disability worldwide (1, 2). Currently, the diagnosis of MDD mainly relies on patients’ clinical symptoms and Hamilton Depression Rating Scale (HDRS). Although their diagnostic value in clinical practice should be recognized, relevant limitations should not be ignored, particularly the unavoidable subjectivity and the insufficient reliability, which leads to the insufficient sensitivity and specificity for early-stage MDD screening. Therefore, developing objective and quantifiable diagnostic indicators is essential to enhance the diagnosis of MDD.

The pathophysiological mechanisms of MDD are complex and closely related to the genetic, environmental, neuroendocrine, cardiovascular, and psychosocial factors (3–6). In addition, the important role of certain biomarkers in the pathogenesis of MDD has been confirmed by several studies, such as 5-hydroxytryptamine, cortisol, and brain-derived neurotrophic factor (7–9). Growing evidence suggests that cerebral microvascular dysfunction may facilitate the development of MDD by inducing chronic ischemia in brain tissue (10). Clinically, positron emission tomography and functional magnetic resonance imaging (MRI) have become a necessary addition to the diagnosis and monitoring of MDD patients (11). Nevertheless, the use of these methods for population-level screening is limited by their high cost, invasiveness, long examination times, or low resolution. Therefore, after fully weighing the cost of healthcare, potential physical harm and overdiagnosis, finding a simple, effective, and fast method to identify neurological abnormality and cognitive profiles is essential for comprehensive and personalizing management, optimizing treatment strategies, saving medical resources, and preventing complications in MDD patients.

In recent years, the relationship between MDD and neurodegenerative diseases has received great research interest. In addition, several studies have revealed that patients with MDD have neurodegenerative changes in the central nervous system, with functional and structural damage in multiple regions of the brain (11–14). As an extracranial extension of the cerebral nerve, the retina provides an easily observable window for monitoring neurodegenerative disease. The retina shares similar physiological and anatomical characteristics with the cerebrum, and the alterations of retinal structural and microvascular parameters may reflect similar variation of those in the cerebral or other end organs (15–19). Recently, thinner nerve fiber layer thickness and enlargement of retinal venules have been reported in patients with mental disorders (20–22). However, traditional methods of measuring retinal microvasculature, such as fundus photography and color Doppler imaging, have the limitation of inconvenient operation and low reliability, which results in the inability of these methods to be widely used in MDD.

Optical coherence tomography angiography (OCTA) provides a unique, quantitative, non-invasive method of assessing the retinal structural and microvascular system, and has been one of the fastest growing and most prospective non-invasive indicators in the field of ophthalmology, and even extra-ophthalmology (23). Interestingly, the use of OCTA as the indicator for neurodegenerative diseases, such as cerebrovascular disease, cognitive impairment and schizophrenia (SP), have been widely reported in recent years (24–27). However, the relationship between MDD and the retinal vasculature remains controversial.

To address these questions, our study enrolled 78 patients with MDD and 47 healthy controls (HCs) to quantitatively evaluate the retinal vessel density and structure changes in patients with MDD by OCTA, and then screen out more reliable retinal indicators for MDD patients through a strict research design. Furthermore, a strong, innovative retina-based diagnostic model for early detection of MDD has been further developed and verified.

## Materials and Methods

### Participants

A group of newly diagnosed and unmedicated MDD patients aged 17 to 55 years in the department of psychiatry, the First Affiliated Hospital of Jinan University from June 2020 and December 2021 were enrolled in this cross-sectional study. Each participant was diagnosed by experienced psychiatrists using the Diagnostic and Statistical Manual of Mental Disorders, 5th Edition (DSM-5) MDD diagnostic standards. In addition, patients with MDD were medication naïve at the time of the enrollment.

All participants were assessed for clinical status using the Young Mania Scale (Y-MRS) and the 24-item HDRS. Patients diagnosed with MDD with a total Y-MRS score < 7 and a total 24-item HDRS score > 20 were able to participate in this study. Exclusion criteria include: (1) a history of major physical or neurological diseases; (2) a history of head trauma; (3) comorbidity with other psychiatric disorders such as anxiety disorders, bipolar disorder (BD), or SP; (4) the history of vasoactive drugs; (5) diabetes mellitus, hypertension or cardiovascular disease; and (6) any history of eye disease, including cataracts, glaucoma, high myopia, macular degeneration, or other diseases that could affect the retinal vessels.

A group of volunteers with 24-item HDRS total score ≤ 7 were included as HCs based on sex-age matching at a ratio of about 1.5 to 1.0. And they were careful screened with the Structured Clinical Interview for DSM-IV Non-patient Edition. Of note, those with mental illness of their own or first degree relatives or with substance abuse were excluded.

Ethics committee approval for this study was obtained from the First Affiliated Hospital of Jinan University, China. Informed consent was given by all participants in this study.

### Basic Ophthalmic Examination

For patients diagnosed with MDD, eye examinations were completed on the day before commencement of the medication. Both the controls and MDD patients were evaluated by the same ophthalmologist who was not known whether they were controls or MDD patients. General ophthalmic exams were performed on each participant, including slit-lamp, fundus examination, intraocular pressure (IOP) examination, best-corrected visual acuity (BCVA) measurement. Visual field examination (Carl Zeiss Meditec, Jena, Germany) was also given to the MDD patients and the HCs, including the mean deviation (MD) and pattern standard deviation (PSD). The visual field results were considered unreliable if the rate of fixation losses is higher than 20%, the false negatives rate is higher than 33%, or the false positives rate is higher than 15%.

### Optical Coherence Tomography Angiography and Optical Coherence Tomography Examination

**FIGURE 1:**
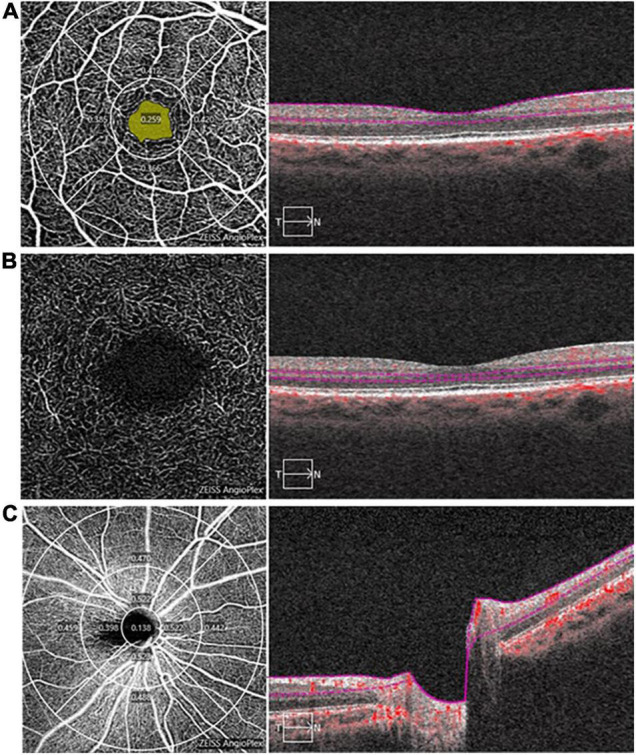
Image analysis of optical coherence tomography angiography (OCTA). **(A)** The superficial capillary plexus (SCP) map in the macula (3 mm × 3 mm), a 1 mm diameter circle and a 3 mm diameter circle divide the SCP into a central area and an inner-circle area, the yellow area is foveal avascular zone (FAZ), the right panel shows a typical segmentation from the inner limiting membrane to the inner plexiform layer; **(B)** The deep capillary plexus (DCP) map in the macula (3 mm × 3 mm), the right panel shows a typical segmentation from the inner nuclear layer to the outer plexiform layer; and **(C)** The radial peripapillary capillary (RPC) map (6 mm × 6 mm), 1, 3, and 6 mm diameter circle divide the RPC into a central, inner-circle and outer-circle area, the right panel shows a typical segmentation of optic nerve head from the inner limiting membrane to the inner plexiform layer.

OCTA examinations were performed by the same examiner using the same equipment (Carl Zeiss Meditec, Dublin, CA, United States). After dilating the pupil, a 3 mm × 3 mm scanning mode was used to acquire macular vascular images and a 6 mm × 6 mm scanning mode was used to acquire optic nerve head vascular images. The technique and principles of OCTA have been described in detail in the previous studies (28, 29). The effects of eye movements and projection-related artifacts were minimized by the OCTA algorithm and eye tracker. We used the OCTA’s built-in software to automate and segment the retinal slabs. The retinal layer segmentation was checked by the experienced ophthalmologist, manual adjustment was performed if necessary. The radial peripapillary capillary (RPC) image in the optic papilla region was segmented automatically by self-contained software. Besides, the retina in macular region was segmented into the four layers: choroidal capillary plexus, avascular layer, deep capillary plexus (DCP), and superficial capillary plexus (SCP). However, due to the lack of blood vessels in the avascular layer and the complex structure of the choroidal capillary layer, we only analyzed the vessel density of RPC (RPC-VD), SCP (SCP-VD), and DCP (DCP-VD) in this study (Figure 1). Since the OCTA device in our study was unable to calculate DCP-VD directly, the DCP images were exported to ImageJ (National Institutes of Health) and binarized to automatically calculate DCP-VD. Vessel density (VD) is a definition of retinal vascular area as a percentage of total area, ranging from 0% (non-perfusion) to 100% (full perfusion). In addition, the foveal avascular zone (FAZ) of the retina was also quantitatively analyzed, including the perimeter (mm), area (mm^2^), and acircularity index (unitless, ranging from 0 to 1). All images were independently reviewed by two experienced fundus specialists. Poor quality images with significant motion artifacts or signal intensity index less than 8 were excluded, and automatic segmentation errors were corrected manually if necessary.

The retinal nerve fiber layer thickness (RNFLT), subfoveal choroidal thickness (SFCT), macular thickness (MT), and ganglion cell complex thickness (GCCT) were evaluated and calculated using the OCT automated software of the same device. The definition of SFCT was the thickness from the inner boundary of the choroid-sclera interface to the retinal pigment epithelium. The retinal nerve fiber layer (RNFL) was divided into four quadrants (temporal, inferior, nasal, and superior), and the average RNFLT and the thickness of each quadrant were assessed.

### Sample Size

We used independent-sample *t* test of PASS 16.0 software (NCSS LLC, East Kaysville, UT, United States) to calculate the minimum sample size by setting the β-value to 0.1 and the α-value to 0.05. The sample size was pre-estimated from our pre-experiment. According to our pre-experimental study, the mean vessel density of HCs and MDD patients were 37.13 and 35.18%, and the standard deviation of the subjects was 2.50%. Therefore, based on a ratio of 1.5 to 1, the minimum sample size of MDD patients and HCs were 45 and 30 after calculation. Furthermore, we assumed that the rate of case exfoliation was 10%, so the minimum sample size should be no less than 50 in MDD group and 33 in HCs after correction.

### Statistical Analysis

Continuous variables between the two groups were compared by independent-sample *t* test. Categorical variables between the two groups were compared by the *chi*-squared test. Bonferroni correction was applied to account for the comparison of OCTA and OCT parameters between the MDD group and the HCs, and the threshold for significance was set to *p* < 0.0025 (adjusted α = 0.0025, 0.05/20). LASSO penalized logistic regression analysis was used to choose the optimal retinal parameters set based on lambda.min and tenfold cross-validation using the R package “glmnet.” Then, the retinal parameters screened above were incorporated into the model of multivariate logistic regression, and diagnostic model was constructed after calculating their coefficients (β values). The diagnostic efficacy of the diagnostic model based on the retina for MDD was evaluated by area under the curve (AUC) of receiver operating characteristic (ROC) curves. In addition, we use the R package “rms” to build a handy nomogram for visualizing the diagnostic model. We used SPSS 22.0 for statistical analysis and GraphPad Prism 8.0.2 for drawing. Differences were regarded as statistically significant with a *p* value < 0.05.

## Results

### Demographic, Clinical and Ocular Characteristics of Participants

Ninety-four patients with MDD were finally included in our study, among whom 11 patients were excluded for poor image quality and 5 were excluded for retinopathy. Therefore, a total of 156 eyes of 78 patients with MDD and 94 eyes of 47 age- and sex-matched HCs were included in this study. As Table 1 shows, there was no remarkable difference between the MDD group and the HCs in sex, age, and education level. The mean duration of disease in MDD patients was 12.23 ± 7.47 months. In addition, the 24-item HDRS score was significantly higher in MDD patients than that in HCs (*p* < 0.001).

**TABLE 1:** Demographic, clinical, and ocular characteristics of participants.

Variables	HCs	MDD	t/χ^2^	*p*-value
Number of participants	47	78		
Sex (male/female)	14/33	27/51	0.310	0.578
Age (year)	24.34 ± 6.67	23.08 ± 5.72	1.122	0.264
Education (year)	15.12 ± 3.99	14.55 ± 2.63	0.880	0.382
Duration of illness (month)		12.23 ± 7.47		
HDRS score	0.95 ± 0.85	26.04 ± 3.94	–54.011	<0.001
Y-MRS score	0.00 ± 0.00	0.86 ± 1.23	–6.143	<0.001
**Ocular characteristics**				
Asthenopia, *n* (%)	9 (19.14%)	13 (16.66%)	0.125	0.724
Diplopia, *n* (%)	3 (6.38%)	4 (5.12%)	0.087	0.768
Eye dryness, *n* (%)	5 (10.63%)	21 (26.92%)	4.721	0.030
BCVA (logMAR)	–0.005 ± 0.019	–0.004 ± 0.017	–0.416	0.677
IOP (mmHg)	14.74 ± 2.25	15.09 ± 2.25	–1.173	0.242
SE (*D*)	–2.27 ± 2.10	–2.10 ± 2.13	–0.637	0.525
Axial length (mm)	24.24 ± 0.88	24.30 ± 0.91	–0.472	0.637
Visual field MD (dB)	–0.52 ± 1.16	–1.91 ± 1.57	8.007	<0.001
Visual field PSD (dB)	1.69 ± 0.64	2.09 ± 0.66	–4.682	<0.001

*HCs, healthy controls; MDD, major depressive disorder; HDRS, 24 item Hamilton Depression Rating Scale; Y-MRS: Young Manic Rating Scale; BCVA, best-corrected visual acuity; IOP, intraocular pressure; SE, spherical equivalent; MD, mean deviation; and PSD, pattern standard deviation.*

No significant differences were found between the MDD group and the HCs in BCVA, IOP, SE, or axial length. The prevalence of eye dryness was markedly higher in the MDD group than that in the HCs (*p* = 0.030). On visual field comparison, MD and PSD were significantly worse in MDD patients (MD, –1.91 ± 1.57 dB; PSD, 2.09 ± 0.66 dB) than those in HCs (MD, –0.52 ± 1.16 dB; PSD, 1.69 ± 0.64 dB; all *p* < 0.001).

### Comparison of Optical Coherence Tomography Angiography and Optical Coherence Tomography Parameters Between Major Depressive Disorder Group and Healthy Controls

The OCTA and OCT parameters of participants are shown in Table 2. The mean, central and inner-circle SCP-VD were 35.86 ± 2.33, 17.14 ± 4.45, and 38.17 ± 2.26% in MDD group and 37.90 ± 1.53, 18.46 ± 5.04, and 40.25 ± 1.58% in the HCs, respectively. The SCP-VD in patients with MDD was significantly lower than that in HCs (*p* < 0.05, uncorrected). In addition, the DCP-VD in MDD group (33.97 ± 1.25%) was significantly lower than that in the HCs (34.73 ± 1.24%; *p* < 0.05, uncorrected). As for the RPC-VD, although the central, inner-circle or mean RPC-VD were not significantly different between the MDD group and the HCs (all *p* > 0.05), the RPC-VD in the outer-circle of the MDD group was remarkably lower compared to the HCs (*p* < 0.05, uncorrected). Interestingly, the FAZ was more irregular in MDD patients than that in HCs (*p* = 0.013, uncorrected), while the FAZ area and perimeter were not significantly different between the groups (all *p* > 0.05). After Bonferroni correction with the threshold at *p* < 0.0025 (adjusted α = 0.0025, 0.05/20), we still found the mean SCP-VD, DCP-VD, and outer-circle RPC-VD were significantly lower in the MDD group as compared to the HCs (all *p* < 0.001).

**TABLE 2:** Comparison of OCTA and OCT parameters between MDD group and healthy controls.

Variables	HCs	MDD	*t*	*p*-value
**SCP-VD (%)**				
Mean	37.90 ± 1.53	35.86 ± 2.33	8.348	<0.001*
Central	18.46 ± 5.04	17.14 ± 4.45	2.157	0.032
Inner-circle	40.25 ± 1.58	38.17 ± 2.26	8.482	<0.001*
DCP-VD (%)	34.73 ± 1.24	33.97 ± 1.25	4.708	<0.001*
**RPC-VD (%)**				
Mean	46.02 ± 2.61	45.47 ± 3.68	1.280	0.202
Central	18.49 ± 10.80	18.04 ± 12.84	0.283	0.778
Inner-circle	47.82 ± 3.78	48.01 ± 3.49	–0.407	0.684
Outer-circle	47.47 ± 2.33	45.60 ± 3.88	4.757	<0.001*
**FAZ**				
Area (mm^2^)	0.28 ± 0.11	0.27 ± 0.09	0.895	0.372
Perimeter (mm)	2.27 ± 0.42	2.21 ± 0.35	1.075	0.284
Acircularity index	0.71 ± 0.06	0.68 ± 0.07	2.512	0.013
**MT (μ m)**				
Central	242.15 ± 16.95	244.24 ± 16.59	–0.954	0.341
Mean	280.34 ± 13.71	279.97 ± 11.99	0.221	0.825
GCCT (μm)	82.60 ± 4.56	82.91 ± 4.83	–0.495	0.621
**RNFLT (μ m)**				
Mean	104.12 ± 9.56	105.06 ± 12.03	–0.642	0.522
Superior	124.96 ± 17.96	121.51 ± 17.60	1.491	0.137
Nasal	72.76 ± 11.89	69.10 ± 11.24	2.437	0.016
Inferior	128.40 ± 19.28	129.83 ± 19.27	–0.568	0.571
Temporal	89.32 ± 15.95	91.58 ± 19.27	–0.953	0.341
SFCT (μm)	259.85 ± 47.54	220.79 ± 44.85	6.520	<0.001*

HCs, healthy controls; MDD, major depressive disorder; SCP-VD, superficial capillary plexus vessel density; DCP-VD, deep capillary plexus vessel density; RPC-VD, radial peripapillary capillary vessel density; FAZ, foveal avascular zone; MT, Macular thickness; GCCT, ganglion cell complex thickness; RNFLT, retinal nerve fiber layer thickness; and SFCT, subfoveal choroidal thickness. *p < 0.0025 significant (with Bonferroni correction, adjusted α = 0.0025, 0.05/20).

As for retinal structure parameters, no statistical differences were found in MT or GCCT between the two groups (all *p* > 0.05). As for the RNFL analysis, the mean RNFLT and the RNFLT of the temporal, inferior and superior sides were not statistically significant between MDD patients and HCs (all *p* > 0.05), except for the nasal RNFLT (*p* < 0.05, uncorrected). Furthermore, the difference in SFCT between the MDD groups and the HCs was significant (*p* < 0.05, uncorrected). After Bonferroni correction with the threshold at *p* < 0.0025 (adjusted α = 0.0025, 0.05/20), we still found the SFCT were significantly thinner in the MDD group as compared to the HCs (*p* < 0.001).

### Identification and Visualization of the Diagnostic Value of Retinal Parameters in Patients With Major Depressive Disorder

**FIGURE 2:**
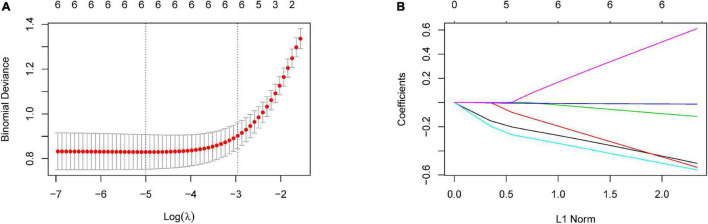
Selection of optimal retinal parameters for modeling by LASSO penalized logistic regression analysis. **(A)** The optimal set of retinal parameters was selected by tenfold cross-validation and lambda.min. **(B)** LASSO coefficient profile of retinal parameters.

According to the remarkably different retinal parameters described above (*p* < 0.001), a combination of six differential retinal parameters was suggested for optimal modeling after LASSO penalized logistic regression analysis, including SCP-VD, DCP-VD, outer-circle RPC-VD, SFCT, MD, and PSD (Figure 2). The coefficients were calculated using multivariate logistic regression analysis, a diagnostic score based on retinal parameters were established (Table 3). Thus, the diagnostic score = (–0.511 × *Z*-score of mean SCP-VD) + (–0.547 × *Z*-score of DCP-VD) + (–0.118 × *Z*-score of outer-circle RPC-VD) + (–0.013 × *Z*-score of SFCT) + (–0.564 × *Z*-score of MD) + (0.624 × *Z*-score of PSD).

**TABLE 3:** Logistic regression model analyses of associations between the retinal parameters and MDD.

Variables	Univariate analysis		Multivariate analysis Coefficient (β)
	OR (95% CI)	*p*-value	
SCP-VD	0.568 (0.475, 0.679)	<0.001	–0.511
DCP-VD	0.605 (0.482, 0.758)	<0.001	–0.547
Outer-circle RPC-VD	0.819 (0.741, 0.907)	<0.001	–0.118
SFCT	0.982 (0.976, 0.988)	<0.001	–0.013
MD	0.503 (0.406, 0.623)	<0.001	–0.564
PSD	2.780 (1.753, 4.407)	<0.001	0.624

*MDD, major depressive disorder; OR, odd ratio; CI, confidence interval; SCP-VD, superficial capillary plexus vessel density; DCP-VD, deep capillary plexus vessel density; RPC-VD, radial peripapillary capillary vessel density; SFCT, subfoveal choroidal thickness; MD, mean deviation; and PSD, pattern standard deviation.*

**FIGURE 3:**
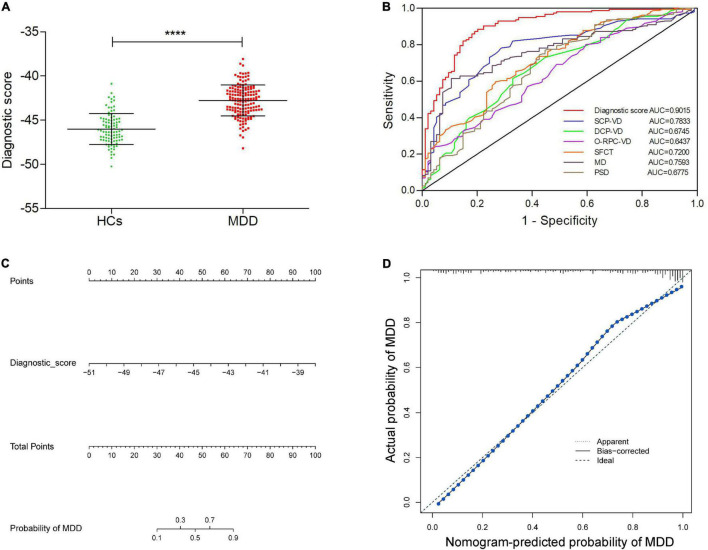
Identification of the value of retina-based diagnostic scores, and establishment and validation of a visual nomogram. **(A)** Comparison of diagnostic scores between MDD group and HCs. **(B)** ROC curves exhibiting the diagnostic efficacy of the retina-based diagnostic score and the six retinal parameters alone. **(C)** The nomogram based on the retina-based diagnostic score for MDD. **(D)** The calibration curve of the nomogram. ****p < 0.0001. HCs, healthy controls; MDD, major depressive disorder; SCP-VD, superficial capillary plexus vessel density; DCP-VD, deep capillary plexus vessel density; O-RPC-VD, outer-circle radial peripapillary capillary vessel density; SFCT, subfoveal choroidal thickness; MD, mean deviation; and PSD, pattern standard deviation.

As show in Figure 3A, the diagnostic scores of MDD patients were significantly higher than those of HCs (*p* < 0.001). ROC analysis further confirmed that the retinal vessel density had a good diagnostic value in MDD patients (SCP-VD, AUC = 0.7833, *p* < 0.001; DCP-VD, AUC = 0.6745, *p* < 0.001; and outer-circle RPC-VD, AUC = 0.6437, *p* < 0.001). In addition, ROC curve analysis also showed that SFCT and visual field had excellent predictability (SFCT, AUC = 0.7200, *p* < 0.001; MD, AUC = 0.7593, *p* < 0.001; and PSD, AUC = 0.6775, *p* < 0.001). Surprisingly, ROC analyses showed that the model had good discriminability for MDD, which was higher than each of the retinal parameters (AUC = 0.9015, *p* < 0.001; Figure 3B). In addition, we have developed a visualized nomogram based on retinal scores in order to strengthen the model’s applicability (Figure 3C). Furthermore, the calibration plot showed a high coherence between the predicted and actual MDD probability, suggesting the nomogram has good diagnostic efficacy for MDD patients (Figure 3D).

## Discussion

The retina is the only visualized neurovascular tissue in the body, providing an easy-to-see window for monitoring systemic neurovascular conditions (30, 31). OCTA is a novel, non-invasive and quantitative retinal imaging tool of which use has been extended from early screening and monitoring of fundus diseases to systemic diseases (32–35). OCTA-base retinal parameters, such as retinal vessel density and thickness, are expected to be one of the most promising and competitive non-invasive indicators in clinical practice.

As a neurodegenerative disease, the diagnosis of MDD mainly relies on the patient’s subjective assessment and lacks quantifiable objective indicators. Although previous studies have demonstrated the structural alterations of the retina in MDD patients, such as thinner RNFLT (36), reduced minimum ganglion cell-inner plexiform layer and poor visual function (37), a highly reliable retinal-related indicators for screening of early-stage MDD remains to be identified for the following reasons. First, previous research indicators on the retina of MDD patients are relatively single, mostly focusing on retinal thickness, and a comprehensive and systematic analysis of the retina is lacking. Furthermore, most of the previous studies had relatively small sample sizes; therefore, the relevant results were not very reliable. Finally, the accuracy of individual retinal parameters is generally limited, hinting at the need to build diagnostic models based on multiple retinal parameters to improve screening capabilities.

Given the above considerations, we performed a comprehensive comparative analysis of retinal parameters between MDD patients and HCs. We found that MDD patients had lower retinal vessel density, thinner SFCT, and poorer visual function compared to HCs. Recently, abnormalities in retinal vessel density and structure have also been demonstrated in psychiatric disorders, such as BD and SP (26, 27, 38–40). As for MDD, although there is no definite evidence directly proving the association between MDD and retinal vessel density, retinal vascular dysfunction and endothelial damage in MDD patients have been confirmed in previous studies (41, 42). Furthermore, the inflammatory hypothesis of MDD may also partially explain our findings. Interestingly, increased inflammatory cells and acute phase proteins have been demonstrated in the cerebrospinal fluid and peripheral blood of MDD patients (43, 44). Inflammatory processes have been demonstrated in several psychiatric disorders, such as SP and depression (45). The inflammatory process may trigger microvascular thrombosis and endothelial inflammation, which may lead to a reduced retinal blood supply and blood flow velocity (46). In addition, compared to other tissues with collateral vessels, the retinal plexus consists of terminal arteries without the anastomotic connections and is more affected by hypoperfusion (47). Retinal and choroidal vascular abnormalities in MDD patients may lead to insufficient supply of nutrients to the retina/choroid, resulting in choroidal thickness changes and visual field damage. Thus, the above factors may contribute to explain the underlying pathophysiological mechanisms of retinal morphological functional impairment in MDD patients in our study.

Moreover, to enhance the accuracy of the above retinal parameters in MDD screening, we developed a new retina-based diagnostic score by appropriate statistical tools, including SCP-VD, DCP-VD, outer-circle RPC-VD, SFCT, MD, and PSD. The results strongly indicated that our model may have powerful diagnostic capabilities for early-stage MDD, which was better than each of the individual retinal parameters. More importantly, based on the diagnostic score, a corresponding visual nomogram was established to enhance its clinical applicability. Consistent with our findings, alterations of above six retinal parameters in psychiatric disorders and their potential clinical roles or pathophysiological impact were more or less revealed in earlier researches (27, 38, 39). To some extent, the retinal parameters mentioned above may be closely related to the development of MDD, which suggests that the corresponding morphological and functional changes in the retina may mirror the pathophysiological alterations of MDD in a timely and reasonable manner.

Given the promising performance of the retina-based model score for MDD diagnosis, this model may provide a valuable guidance for early screening and efficacy assessment of MDD. On the one hand, some MDD patients usually conceal their condition from others out of fear of public rejection, thus further prolonging their suffering. For the screening of these high-risk groups, relying on subjective symptoms and depression scale scores alone is often prone to underdiagnosis and misdiagnosis, which may result in serious consequences. Therefore, the use of retina-related artificial intelligence models for early screening of people at high risk of MDD and targeted specialty evaluation and treatment of patients with suspected MDD may be helpful for reducing healthcare costs and self-harm or suicide due to underdiagnosis. On the other hand, with further in-depth research, retina-base model scoring of MDD patients at different stages of the treatment process may be useful in assessing efficacy, guiding treatment and predicting prognosis. However, the transition from theory to practice still faces many challenges. In the future, with the further development of artificial intelligence, the retina-based MDD diagnostic model will have a broader clinical application prospects.

There are still some limitations of this study in spite of the promising results. First, the sample size of this cross-sectional study is small, the effects of disease severity and duration on the retina have not been adequately observed. In addition, this study mainly observed patients with unmedicated depression, and the effect of antidepressant on retinal blood vessels and structures are unclear, which needs to be confirmed by a further follow-up study. Furthermore, although the diagnostic accuracy of MDD was demonstrated in our study, its capability to identify early MDD needs a further confirmation in large prospective studies and validation with external data to get additional efficacy evidence. Moreover, future studies could perform comparative analysis of retinal parameters in multiple psychiatric disorders to confirm the specificity of retinal changes in different psychiatric disorders. Finally, the direct relationship between cerebral vascular lesions on MRI and retinal vessel density using OCTA in patients with MDD requires a more systematic study to confirm.

## Conclusion

In conclusion, based on a rigorous study design and reliable data, we identified a unique set of MDD-related retinal parameters. We found that patients with MDD have lower retinal vessel density, thinner retinal structure and poor visual function. Moreover, a credible diagnostic score based on the retina and corresponding visualized nomogram were developed by a machine learning approach. Undoubtedly, the role of a patient’s clinical symptoms and HDRS score in MDD diagnosis is irreplaceable. However, with the development of artificial intelligence technology and interdisciplinary collaboration, the retina-based model may become a promising alternative for early detection of MDD in clinical practice, thus ensuring the best timing for early detection and treatment.

## Data Availability Statement

The raw data supporting the conclusions of this article will be made available by the authors, without undue reservation.

## Ethics Statement

The studies involving human participants were reviewed and approved by Ethics Committee of the First Affiliated Hospital of Jinan University. Written informed consent to participate in this study was provided by the participants’ legal guardian/next of kin.

## Author Contributions

JZ, YJ, XL, and LL: general study design and drafting the manuscript. XL and SL: worked on data acquisition. XL: writing original draft and writing review and editing. GY, HY, JZ, LL, SL, SM, SZ, XL, and YJ: critical revision of the manuscript. All authors contributed to the article and approved the submitted version.

## Conflict of Interest

The authors declare that the research was conducted in the absence of any commercial or financial relationships that could be construed as a potential conflict of interest.

## Publisher’s Note

All claims expressed in this article are solely those of the authors and do not necessarily represent those of their affiliated organizations, or those of the publisher, the editors and the reviewers. Any product that may be evaluated in this article, or claim that may be made by its manufacturer, is not guaranteed or endorsed by the publisher.
